# Experimental promoter identification of a foodborne pathogen *Salmonella enterica* subsp. *enterica* serovar Typhimurium with near single base-pair resolution

**DOI:** 10.3389/fmicb.2023.1271121

**Published:** 2024-01-04

**Authors:** Sang-Mok Lee, Hoa Thi Le, Assiya Taizhanova, Linh Khanh Nong, Joon Young Park, Eun-Jin Lee, Bernhard O. Palsson, Donghyuk Kim

**Affiliations:** ^1^School of Energy and Chemical Engineering, Ulsan National Institute of Science and Technology (UNIST), Ulsan, Republic of Korea; ^2^Department of Genetic Engineering and Graduate School of Biotechnology, College of Life Sciences, Kyung Hee University, Yongin, Republic of Korea; ^3^Department of Life Sciences, College of Life Sciences and Biotechnology, Korea University, Seoul, Republic of Korea; ^4^Department of Bioengineering, University of California San Diego, La Jolla, CA, United States

**Keywords:** *Salmonella enterica* serovar Typhimurium LT2, promoter, sigma factor network, ChIP-exo, RNA-seq

## Abstract

*Salmonella enterica* serovar Typhimurium (*S.* Typhimurium) is a common foodborne pathogen which is frequently used as the reference strain for *Salmonella*. Investigating the sigma factor network and protomers is crucial to understand the genomic and transcriptomic properties of the bacterium. Its promoters were identified using various methods such as dRNA-seq, ChIP-chip, or ChIP-Seq. However, validation using ChIP-exo, which exhibits higher-resolution performance compared to conventional ChIP, has not been conducted to date. In this study, using the representative strain *S.* Typhimurium LT2 (LT2), the ChIP-exo experiment was conducted to accurately determine the binding sites of catalytic RNA polymerase subunit RpoB and major sigma factors (RpoD, RpoN, RpoS, and RpoE) during exponential phase. Integrated with the results of RNA-Seq, promoters and sigmulons for the sigma factors and their association with RpoB have been discovered. Notably, the overlapping regions among binding sites of each alternative sigma factor were found. Furthermore, comparative analysis with *Escherichia coli* str. K-12 substr. MG1655 (MG1655) revealed conserved binding sites of RpoD and RpoN across different species. In the case of small RNAs (sRNAs), 50 sRNAs observed their expression during the exponential growth of LT2. Collectively, the integration of ChIP-exo and RNA-Seq enables genome-scale promoter mapping with high resolution and facilitates the characterization of binding events of alternative sigma factors, enabling a comprehensive understanding of the bacterial sigma factor network and condition-specific active promoters.

## Introduction

*Salmonella enterica* subsp. *enterica* serovar Typhimurium (*S.* Typhimurium) has been known to be the main cause of global human gastroenteritis, and more than 2,600 different serovars have been identified to date (Desai et al., 2013; Jajere, 2019). *Salmonella* Typhimurium LT2 (LT2) is a representative strain for cellular and genetic analysis in *Salmonella* which was first sequenced in 2001 (McClelland et al., 2001). It has become a standard genome for comparative genomic approaches to closely related *Salmonella* or other enterobacteria (Samal et al., 2015; Vila Nova et al., 2019).

In an ever-changing environment, bacterial cells tune their transcriptional programs by regulating the binding and catalytic activity of RNA polymerase (RNAP; Marcus et al., 2000). For them to adapt and reproduce under different extreme conditions during the infection processes, the housekeeping sigma factor RpoD and alternative sigma factors (e.g., RpoN, RpoS, RpoE) have also been found to play critical roles in the regulation of virulence and its associated genes (Kazmierczak et al., 2005). For example, RpoS highly enhances virulence factor activity such as *spv* gene cluster against the host defense system (Andino and Hanning, 2015). In addition to sigma factors, recent studies have discovered that small regulatory RNAs (sRNAs) have been widely studied due to their meaningful regulatory roles in bacteria especially on virulence genes (Lee and Gottesman, 2016; Gao et al., 2017; Ahmed et al., 2019). Similar to transcription factors, sRNAs can interfere or increase ribosome binding, strengthen or weaken mRNA stability, and some even can control the activity of the protein (Storz et al., 2011). Environmental stresses reveal sRNAs to coordinate the adaption processes of a bacterium (Barnhill et al., 2019). For example, sRNAs such as *isrI* were identified their roles when *Salmonella* infected with certain conditions such as low oxygen and low magnesium (Padalon-Brauch et al., 2008; Gong et al., 2011). Given the continuous adaptation of *S.* Typhimurium’s transcriptomic regulation in response to environmental shifts, pinpointing the locations of promoters that operate under specific conditions and understanding their transcriptomic character are pivotal in unraveling the complexity of the transcriptional circuitry involved in regulating the bacterial mechanism.

There have been several attempts to identify promoters with both computational and experimental approaches (Harley and Reynolds, 1987; Wade et al., 2006; Rhodius and Mutalik, 2010; Sharma et al., 2010; Kim et al., 2012; Cho et al., 2014). The identification of promoter elements in the genomic DNA by computational methods depends on the statistical analysis of consensus sequences as overrepresented regions. However, such sequence elements in promoters are not fully conserved in the sequence, thus producing many false-positive predictions (Kim et al., 2012). While experimental methods such as TSS-seq (Kim et al., 2012; Seo et al., 2012; Cho et al., 2014), differential RNA-seq (Sharma et al., 2010), or ROSE (Schmidt et al., 2023) have shown better performance in identifying transcription start sites and their associated promoters, they do not provide information on which sigma factors are associated with those promoters. Chromatin immunoprecipitation (ChIP) is known as the most widely used method to identify genomic binding locations of sequence-specific regulatory proteins. Previous researchers first started with ChIP-Chip and then ChIP-seq by collected big fragment library of DNA (Cho et al., 2014); however, it was hard to detect exact binding regions because the peak resolution of the regions was quite ambiguous approximately 500–1,000 bp. This lack of precision provides a challenge to identify promoters of small proteins or small RNA genes. Since 2010, ChIP combined lambda exonuclease digestion followed by high-throughput DNA sequencing (ChIP-exo) has been investigated with exonuclease treatment to get much tighter resolution and identify accurate binding sites for multiple transcription factors, such as Cra (Kim et al., 2018), Fur (Seo et al., 2014), ArgR (Cho et al., 2015), GadEWX (Seo et al., 2015a), OxyR/SoxR/SoxS (Seo et al., 2015b), OmpR (Seo et al., 2017), and uncharacterized transcription factors (Gao et al., 2018) in *E. coli* str. K-12 substr. MG1655 (MG1655). Thus, it was expected that the same approach might yield detailed knowledge for promoters and sigma factor-binding sites for closely related bacteria such as *Salmonella*. Experimental identification of genome-wide active promoters under a specific condition may be an important reference for studying how *Salmonella* respond to their environment. Thus, this study aims to capture the genome-wide *in vivo* binding sites of major sigma factors in the LT2 and identify active promoters during the exponential phase with near single base-pair resolution employing ChIP-exo and RNA-seq.

## Results

### Genome-scale binding landscape of RNA polymerase subunits

**Figure 1:**
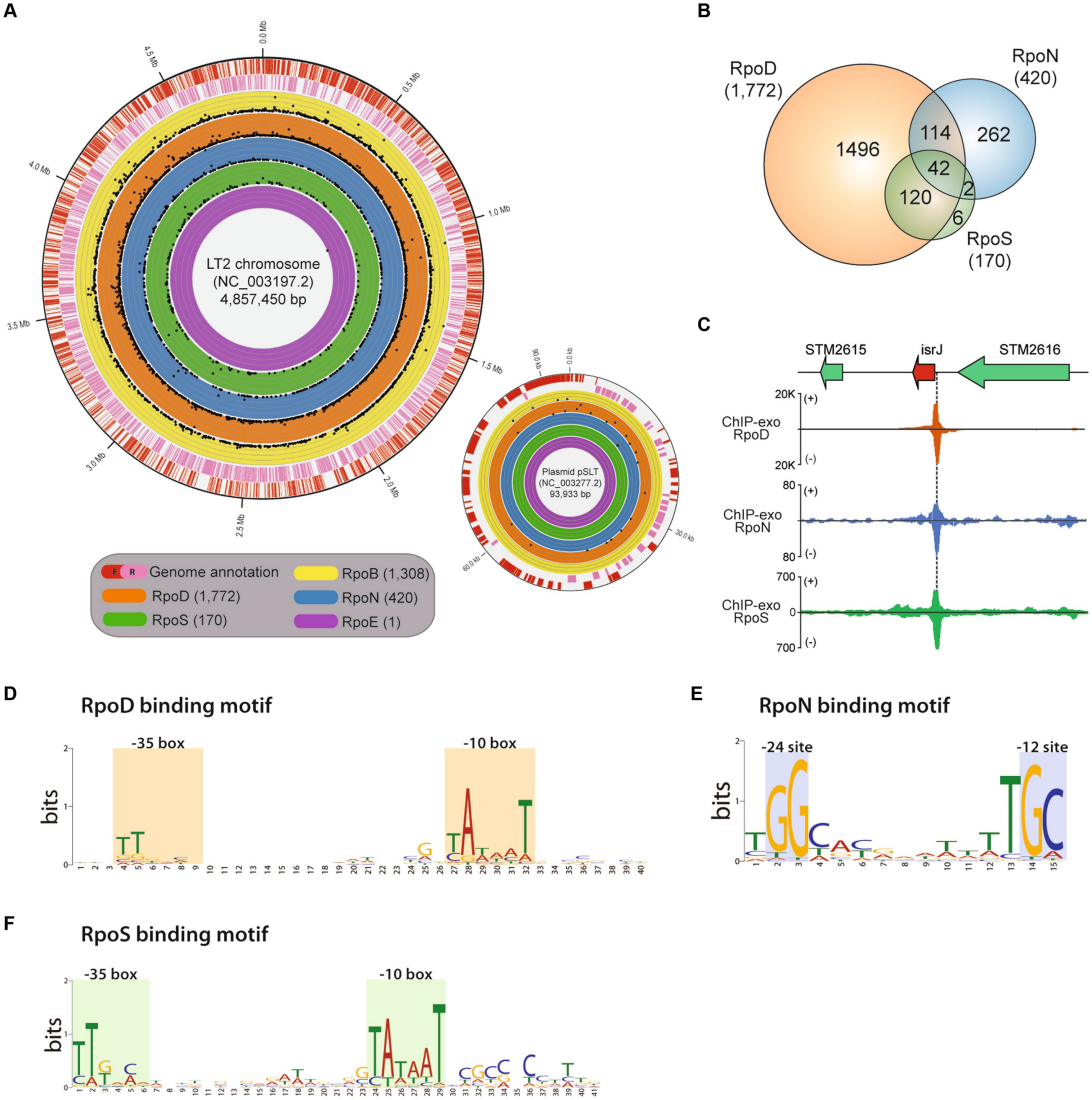
Genome-wide binding landscapes of RNA polymerase subunits in *Salmonella enterica* subsp. *enterica* serovar Typhimurium LT2. **(A)** The circular visualization of the whole genome of the main chromosome and plasmid of LT2 (red—the forward strand, pink—the reverse strand) with binding sites of the catalytic RNA polymerase subunit, RpoB, and 4 sigma factors, RpoD, RpoN, RpoSs, and RpoE. The numbers of identified binding sites for RpoB, RpoD, RpoN, RpoS, and RpoE with ChIP-exo experiments are 1,308, 1,772, 420, 170, and 1, respectively. Each dot represents the binding position and its binding intensity (signal-to-noise ratio) of the sigma factors. **(B)** Distribution of shared binding sites among RpoD, RpoN, and RpoS. **(C)** Zoomed-in example of overlapping binding sites among RpoD, RpoN, and RpoS, directly upstream of a virulent sRNA gene *isrI*. **(D–F)** Sequence motifs for binding sites of three major sigma factors: **(D)** RpoD **(E)**, RpoN, and **(F)** RpoS. Motif search was performed using the MEME suite, with binding motifs calculated from at least 90% of the input sequences obtained by each sigma factor ChIP-exo data.

To determine the genomic location of promoters in the LT2 genome, the genome-wide binding profiles of RNAP and sigma factors were explored using samples obtained from the exponential phase by ChIP-exo with antibodies for RpoB, RpoD, RpoN, RpoS, and RpoE as previously described (Seo et al., 2014; Figure 1A). Under the same condition, RNA-seq was performed as well, and bioinformatic analysis was performed to integrate the ChIP-exo and RNA-seq data.

The investigated binding sites for each transcriptional regulatory element were as follows: RpoB (RNAP), 1,308; RpoD, 1,772; RpoN, 420; RpoS, 170; RpoE, 1 (Figure 1B; Supplementary Table S1). A total of 2,363 binding sites for RpoD, RpoN, RpoS, and RpoE were experimentally identified, directly regulating 2,649 downstream genes. Among the binding sites, 2,192 (92.8%) binding sites were under the regulatory network of either sigma factors RpoD, RpoN, or both. In the case of RpoS, there were 162 (95.3%, 162/170) sites being also co-regulated by RpoD, of which 42 were found to be sites that bind RpoN at the same time. These results substantiate the role of RpoD that is responsible for the transcription of all alternative sigma factors and itself as well.

Subsequently, the characterization of sigma factor-binding sites was conducted. First, overlapping binding sites were defined as any cases where the *in vivo* binding sites of sigma factors overlap even slightly in genomic location. Figure 1C shows the representative example which was located upstream of the sRNA gene—*isrI*. Next, the average width of the binding sites for each sigma factors was also measured as follows: RpoD (40 ± 2 bp), RpoS (40 ± 2 bp), and RpoN (35 ± 2 bp; Supplementary Figure S1). All the binding width distributions exhibited a leptokurtic distribution shape, suggesting that the exonuclease treatment successfully homogenized the length of DNA fragments, ensuring high-quality ChIP-exo data. Sequence motifs of binding sites for each sigma factors RpoD, RpoN, and RpoS were also identified (Figures 1D–F). Although it was reported that the motif of *S.* Typhimurium SL1344 is slightly different from that of *E. coli,* with a stronger “extended”—10 motif (Kröger et al., 2012), another study in 2014 using ChIP-exo demonstrated that RpoD recognizes the −10 (TATAAT) and −35 (TTGACA) boxes promoter elements in *E. coli* (Kim, 2014). In this study, similar to the previous studies, it was found that RpoD (Figure 1D) and RpoS (Figure 1F) of LT2 hold a high conservation motif at −10 and −35 region TTG[20]TataaT which was found in both *E. coli* and *K. pneumoniae* (Kim et al., 2012). The sequence motif tGGCa[7]TGC was obtained for RpoN which is located at −12 and −24 region with respect to the transcription start site in LT2, which was similar to the previously found in MG1655 (Bang et al., 2023a) as well (Figure 1E).

The distribution of RpoB, RpoD, RpoN, and RpoS binding sites was further characterized based on the strand orientation of their respective promoter regions (Table 1). All binding sites were classified into three groups based on their strand specificity as follows: (1) binding to the forward strand promoter region; (2) binding to the reverse strand promoter region; and (3) binding to the divergent promoter region. First, out of 1,308 RpoB binding sites, 42.4% (555/1,308) was located on the forward strand promoters and 40.4% (529/1,308) on the reverse strand promoters, while there was 17.1% (224/1,308) on the divergent promoter regions. In the case of RpoD, the distribution of 1,772 binding sites was observed as well: 40.7% (722/1,772) on the forward strand, 44.2% (784/1,772) on the reverse strand, and 15.0% (266/1,772) on the divergent promoters. Among 420 RpoN binding sites, the more equal allocation was seen on the forward and reverse promoters [forward: 199/420 (47.4%), reverse: 201/420 (47.9%)], whereas only 4.8% (20/418) constituted the binding sites on the bidirectional promoters. Based on the same classification, the RpoS binding sites were distributed as 41.2% (70/170) on the forward, 38.2% (65/170) on the reverse strand, and 20.6% (35/170) on the divergent promoters. Only one binding site on the divergent promoter region was observed for RpoE.

**Table 1:** Binding site distribution of RpoB and sigma factors across different promoter types.

Sigma factors	Position of binding sites	No. binding sites	Forward P	Reverse P	Divergent P
RpoB (1,308)	Intergenic	887	316	348	224
	Intragenic	421	239	181	
RpoD (1,772)	Intergenic	1,304	491	550	266
	Intragenic	468	231	234	
RpoN (420)	Intergenic	114	46	49	20
	Intragenic	304	153	152	
RpoS (170)	Intergenic	110	37	38	35
	Intragenic	60	33	27	
RpoE (1)	Intergenic	1	N.D.	N.D.	1
	Intragenic	0	N.D.	N.D.	

Following strand specificity of downstream target genes: forward promoter (forward P), reverse promoter (reverse P), and divergent promoter (divergent P). N.D. indicates non-detected.

### Characterization of RpoD and RpoN binding events associated with RpoB

As RpoD and RpoN covered more than 99% of genes or transcriptional units in LT2 under the exponential phase, understanding their mode of action may be important to unravel the transcriptional regulatory mechanism of the bacterium. Notably, there were some studies reporting intragenic promoters whose unique features have been identified (Shimada et al., 2008; Bonocora et al., 2015; Fitzgerald et al., 2018). Therefore, it needs to observe the binding sites of each alternative sigma factor separately in intra- or intergenic region.

**Figure 2:**
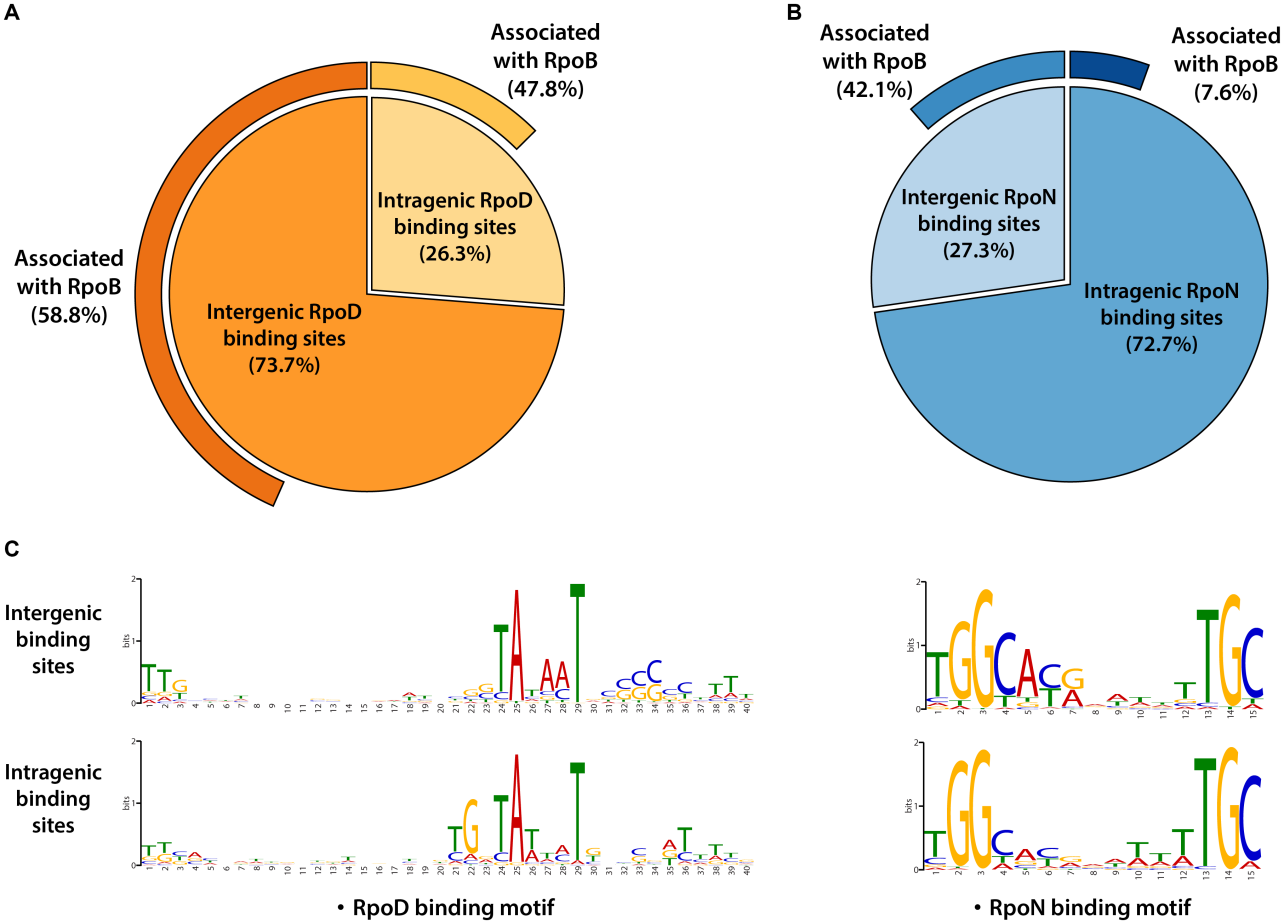
Genomic locations for binding sites of major sigma factors RpoD and RpoN in LT2. **(A)** Distribution of genomic locations for binding sites of RpoD and **(B)** RpoN in pie charts. The outmost curves for each chart indicate the proportions of their association with RpoB binding events. **(C)** The sequence motifs of intergenic and intragenic binding sites for RpoD and RpoN. Motif search was performed using the MEME suite, with binding motifs calculated from at least 90% of the input sequences for each case.

The RpoD and RpoN binding sites were categorized according to whether their locations were intragenic or intergenic ones. RpoD binding sites consisted of 1,292 (73.7%) intergenic binding sites and 460 (26.3%) intragenic binding sites (Figure 2A). RpoN binding sites were composed of 114 (27.3%) intergenic binding sites and 304 (72.7%) intragenic binding sites (Figure 2B). Furthermore, the consensus sequences for each group (intergenic and intragenic for RpoD and RpoN) were analyzed separately to compare the similarity of sequence motifs of the promoter. Interestingly, sequence motifs of intergenic and intragenic binding sites by RpoD and RpoN were identical, respectively (Figure 2C). Consequently, these observations lead to a question about what function intragenic binding sites might have and whether they contribute to a transcription event. To understand more about intragenic binding sites in LT2 and their differences from intergenic ones, further analyses were made with ChIP-exo datasets for RpoB, a catalytic subunit of RNAP complex, to assess genome-wide RpoB binding sites and their binding intensities. Within 1,752 RpoD binding sites, the majority (58.8%, 760/1,292) of intergenic binding sites were associated with RpoB binding site activity, and nearly half (47.8%, 220/460) of intragenic binding sites were also associated with RpoB as well. Interestingly, significantly less overlapping binding events were observed between RpoB and RpoN. Only 7.6% (23/304) of intragenic RpoN binding sites were associated with RpoB, whereas 42.1% (48/114) of intergenic binding sites were overlapped (Figure 2B).

**Figure 3:**
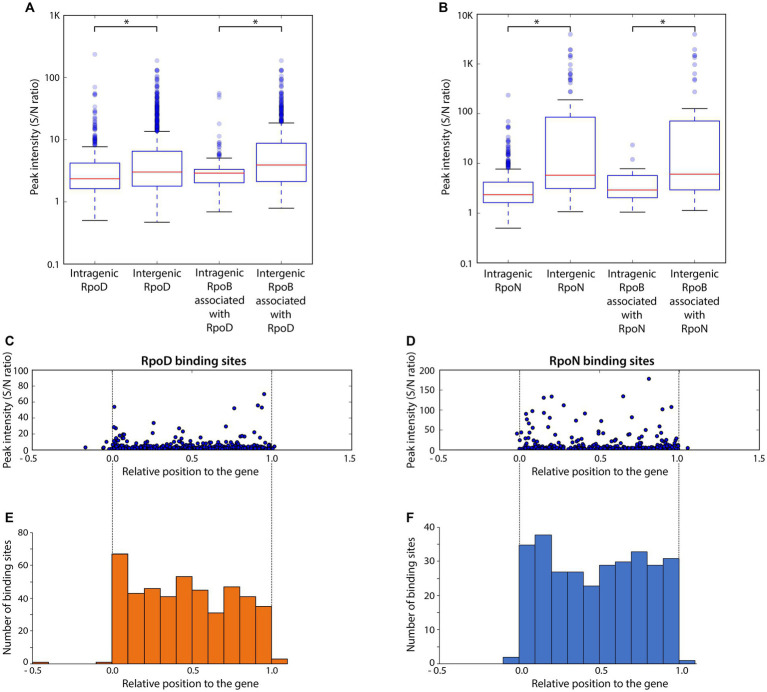
Comparison of binding intensities for intergenic and intragenic binding sites and relative binding locations of intragenic binding sites for RpoD and RpoN. **(A,B)** The boxplots represent the binding intensities of ChIP-exo peaks (signal-to-noise ratio) for intergenic and intragenic binding sites in **(A)** the entire RpoD dataset and the subset associated with RpoB and **(B)** those of RpoN. The background noise level was determined based on the highest 5% of signals at genomic positions. This decision was made because the top 5% of signal intensities, across all ChIP-exo replicates and conditions, align closely with the total number of reads and represent the background level within the plateau. Asterisks in the two box plots indicate significance values (*p* ≤ 0.05) determined by the Mann–Whitney *U*-test. **(C,D)** Distribution of relative binding locations and binding intensities for intragenic binding sites across genes of **(C)** RpoD and **(D)** RpoN. **(E,F)** Frequency of intragenic binding sites for **(E)** RpoD and **(F)** RpoN according to their relative position inside genomic regions.

Binding intensities of alternative sigma factors and RpoB bound at the same sites were compared for RpoD (Figure 3A) and RpoN (Figure 3B). Overall, significant differences on the binding intensities of RpoB between intergenic and intragenic sites were observed in both cases. The intragenic binding sites of both RpoD and RpoN also had a weaker average peak intensity than their intergenic counterparts, respectively. Similarly, for the intensity of RpoB binding sites associated with RpoD or RpoN, intragenic binding sites had weaker binding intensities than intergenic binding sites, suggesting a general preference in RpoB association with intergenic sites over intragenic regions. Among the groups of RpoD and RpoN binding sites, intergenic RpoN revealed the widest spread of peaks with the highest overall binding intensity. Additionally, RpoB-associated RpoN binding sites exhibited a higher peak intensity as compared to the RpoB-associated RpoD binding sites as well. With respect to the position of binding sites within the gene, no bias in binding intensity was observed (Figures 3C,D). Based on the ChIP-exo analysis, the locations of both RpoD and RpoN intragenic binding sites were found to be distributed rather randomly across the genes (Figures 3E,F).

**Figure 4:**
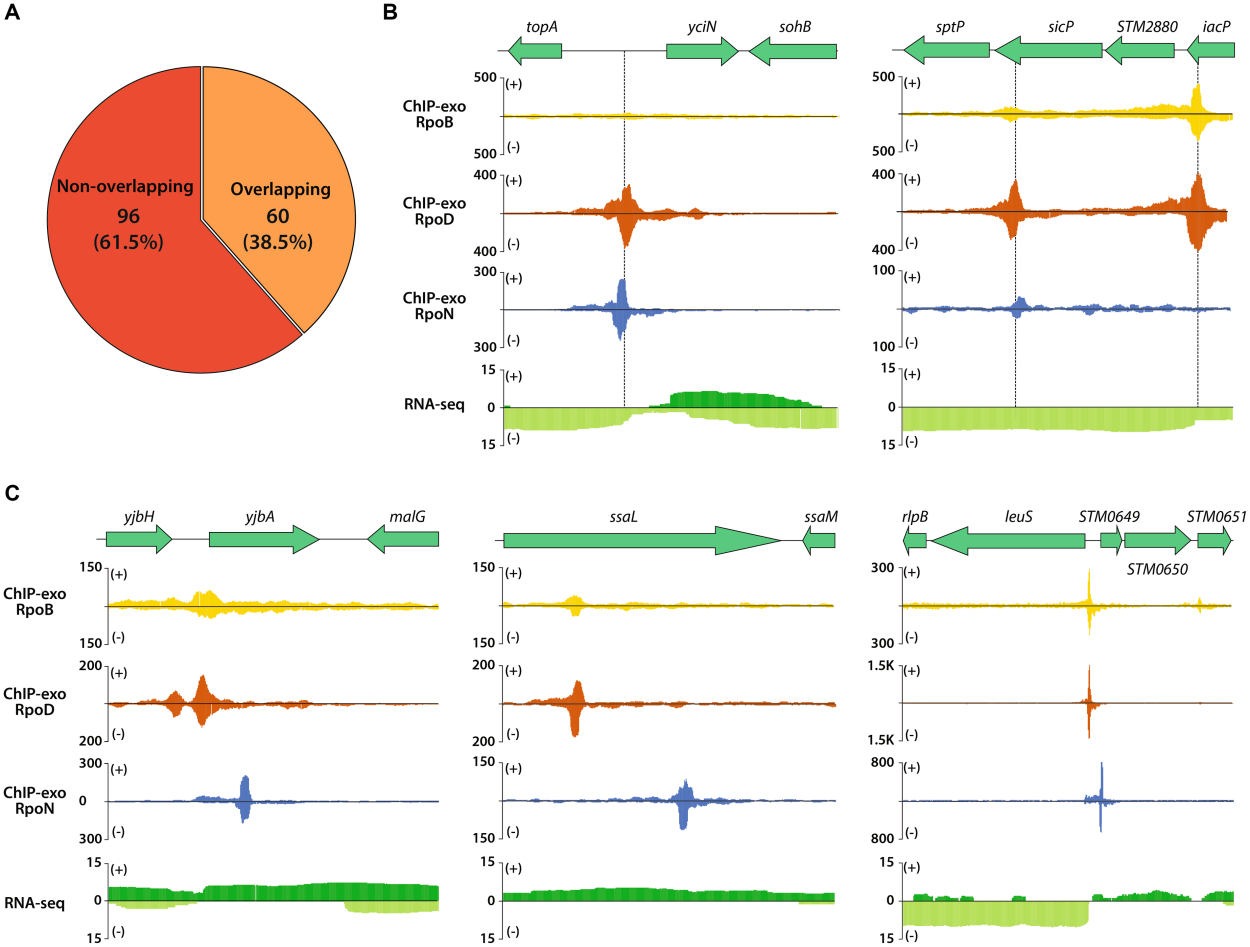
Overlapping binding sites between RpoD and RpoN binding events in the context of RpoB-associated cases. **(A)** Proportion of overlapping binding events between RpoB-associated RpoD and RpoB-associated RpoN. **(B)** Zoomed-in examples displaying overlapped binding events among RpoD and RpoN ChIP-exo datasets integrated with RNA-seq transcriptome dataset in the intergenic region (*topA*) and intragenic region (*sptP* and STM2880). **(C)** Three zoomed-in examples of integrated ChIP-exo and RNA-seq datasets for non-overlapping binding sites for *yjbH*, *ssaL*, and *leuS*.

Meanwhile, the sigma factor-binding map with nearly single base-pair resolution enabled accurate comparison between binding sites of sigma factors even if they were closely located around the same gene, which was rather difficult with previously established ChIP methods such as ChIP-chip or ChIP-seq. The binding sites of RpoB, RpoD, and RpoN could be classified into two types: overlapped (matched) binding regions and non-overlapped (mismatched) binding regions (Figure 4A). In LT2, 60 binding regions were detected overlapped by all the three factors. Figure 4B indicates representative example of the regions. The majority were intergenic sites (53/60), while, among 53 regions, 20 were on the forward strand, 11 were on the reverse, and 22 were on the intergenic divergent promoter regions. There were three main patterns of adjacent peak distribution being observed (Figure 4C). The most prominent case is the sharing of RpoB and RpoD binding regions outside the gene, at promoter regions, while the RpoN binding position is located further within that gene.

### Genome-wide transcriptome feature of LT2

On top of an update on the genome annotation of LT2, a genome-wide transcriptome analysis with strand-specific RNA-seq was performed to delineate the landscape of transcriptome and to ascertain information on transcriptional expression and regulation of the genome. The cDNA reads obtained for the LT2 were 8,864,692 and 10,085,972, with a mapping rate of over 93 and 88%, respectively. Among the 4,451 CDS in the main chromosome and 103 CDS in the plasmid of the LT2 genome, the transcriptome analysis revealed that 2,861 CDS in the main chromosome and 52 CDS in the plasmid were expressed, indicating that 64.0% of the total genes were expressed (with TPM value > 10) under the given growth condition (Supplementary Table S2). While about over 60% of genes of the main chromosome were transcribed, only about 50% of the genes located in the plasmid showed their expression.

Meanwhile, alternative sigma factors such as RpoN regulate directly or indirectly the expression of virulence or virulence-associated genes in Gram-negative pathogens although its role differentiates from species to species (Kazmierczak et al., 2005). A comprehensive search of the open source database VFDB (Liu et al., 2022) has identified a total of 165 genes with virulence properties in the LT2 genome, of which 156 and 9 are located in the chromosome and plasmid, respectively (Supplementary Table S3). When investigating the expression of these virulence-associated genes, it was confirmed that only 26.7% (44/156) of the genes exhibited their expression. The functions of expressed genes included *phoP* and *phoQ* (PhoPQ two-component regulatory system) and *sip* cluster (*Salmonella* invasion proteins cluster; *sipA*, *sipD*, *sipC*, and *sipB*) whose products are reported to be responsible for the secretion and translocation of SPI-1 effectors (Lou et al., 2019). All the ChIP-exo targets RpoD, RpoN, RpoS, and RpoE, which have been reported to be involved in virulence processes (Kazmierczak et al., 2005; Dong and Schellhorn, 2010; Bonocora et al., 2015), also showed their expression (Supplementary Table S2).

### Comparison of sigmulons between *Escherichia coli* and *Salmonella enterica* subsp. *enterica* serovar Typhimurium

With the reconstruction of genome-wide binding maps for RNAP subunits with near single base-pair resolution, it became of interest to investigate how genes were regulated by multiple different sigma factors. The RNA-seq data provide putative transcriptional unit structures for co-transcribed genes. Thus, transcriptome data were integrated with ChIP-exo-mediated sigma factor-binding information and were used to define sigmulons for major sigma factors. Subsequently, distinguished features of the sigmulons in LT2 were investigated by comparative genomic analysis of LT2 with MG1655 which is an anchor strain for comparative genomic analysis on various pathogenic enterobacteria.

**Figure 5:**
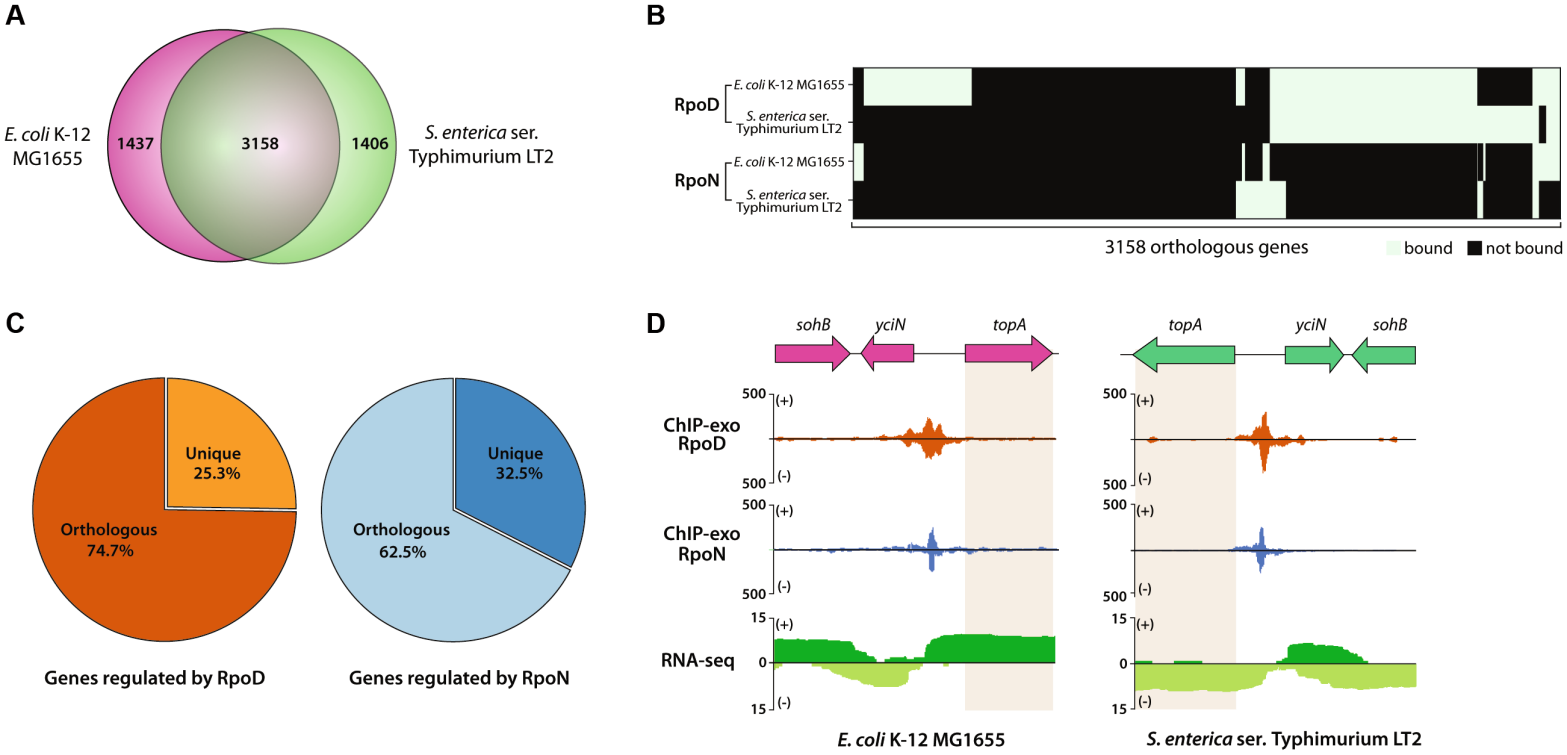
Comparative analysis of RpoD and RpoN sigmulons in *Escherichia coli* str. K-12 substr. MG1655 (MG1655) and LT2. **(A)** Venn diagram for the number of orthologous genes (coding genes and sRNAs) between MG1655 and LT2. **(B)** Heatmap illustrating the binding patterns of RpoD and RpoN for the orthologous genes between MG1655 and LT2. **(C)** The proportion of orthologous genes in sigmulons of RpoD and RpoN. **(D)** Zoomed-in example of promoter identification of *topA*, which is regulated by RpoD and RpoN in both MG1655 and LT2.

First, the genomic contents of two closely related enterobacteria, MG1655 and LT2, were compared (Figure 5A). Two bacteria shared 3,158 genes, corresponding to 70.0% (3,158/4,714 genes) of LT2 and 68.7% (3,158/4,595 genes) of MG1655. The rest of the genes accounting for 30.0% of genes in LT2 includes *Salmonella*-specific genes, such as pathogenic islands, SPI1, and SPI2. Some of the 3,158 orthologous genes were found to be bound by RpoD, of which 284 were LT2-specific, 517 were MG1655-specific, and 983 were common, respectively (Figure 5B). Similarly, 212, 201, and 68 orthologous genes bound by RpoN were LT2-specific, MG1655-specific, or common, respectively. Aside from the orthologs, the numbers of unique genes regulated by each sigma factor were also enumerated, which 25.3% of RpoD-regulated genes (428/1,695) and 32.5% (135/415) of RpoN-regulated genes were specific to LT2 (Figure 5C). Furthermore, there were several orthologs being regulated by both RpoD and RpoN sigma factors in the same manner for both strains. For instance, the binding regions of RpoD and RpoN were identified upstream of *topA*, an essential gene for DNA topoisomerase 1 regulated by RpoD and RpoN for both MG1655 and LT2 (Yamaguchi and Inouye, 2015), which corresponds with its gene expression (Figure 5D).

### Identification of sRNAs and its operational feature in LT2

To investigate the expression patterns of sRNAs in the LT2 genome, we aimed to assemble a comprehensive list of predicted sRNAs. For this purpose, we examined whether sRNA sequences reported in various references were conserved in LT2. Initially, we included non-coding RNAs (ncRNAs) from the NCBI GenBank file (NC_003197.2, NC_003277.2), identifying nine candidates. Next, we utilized the sequence information of 871 sRNAs in the *S.* Typhimurium SL1344 strain from a study that has reported the highest number of sRNAs in the *S.* Typhimurium to date, to determine their presence in the LT2 (Houserova et al., 2021). Additionally, we expanded the list by integrating sRNA information from *E. coli* MG1655, obtained through four different databases as follows: NCBI,^1^ EcoCyC,^2^ BSRD,^3^ and Rfam (Supplementary Table S4). Furthermore, we sought to determine whether any of the *Salmonella* adhesive-associated sRNA (SaaS) sequences reported in the *S. enteritidis* strain NCM61 were also present in LT2 (Cai et al., 2023). We run the Infernal algorithm (Nawrocki et al., 2009) with sRNA models from Rfam database 14.5^4^ to broaden our search as well.

**Figure 6:**
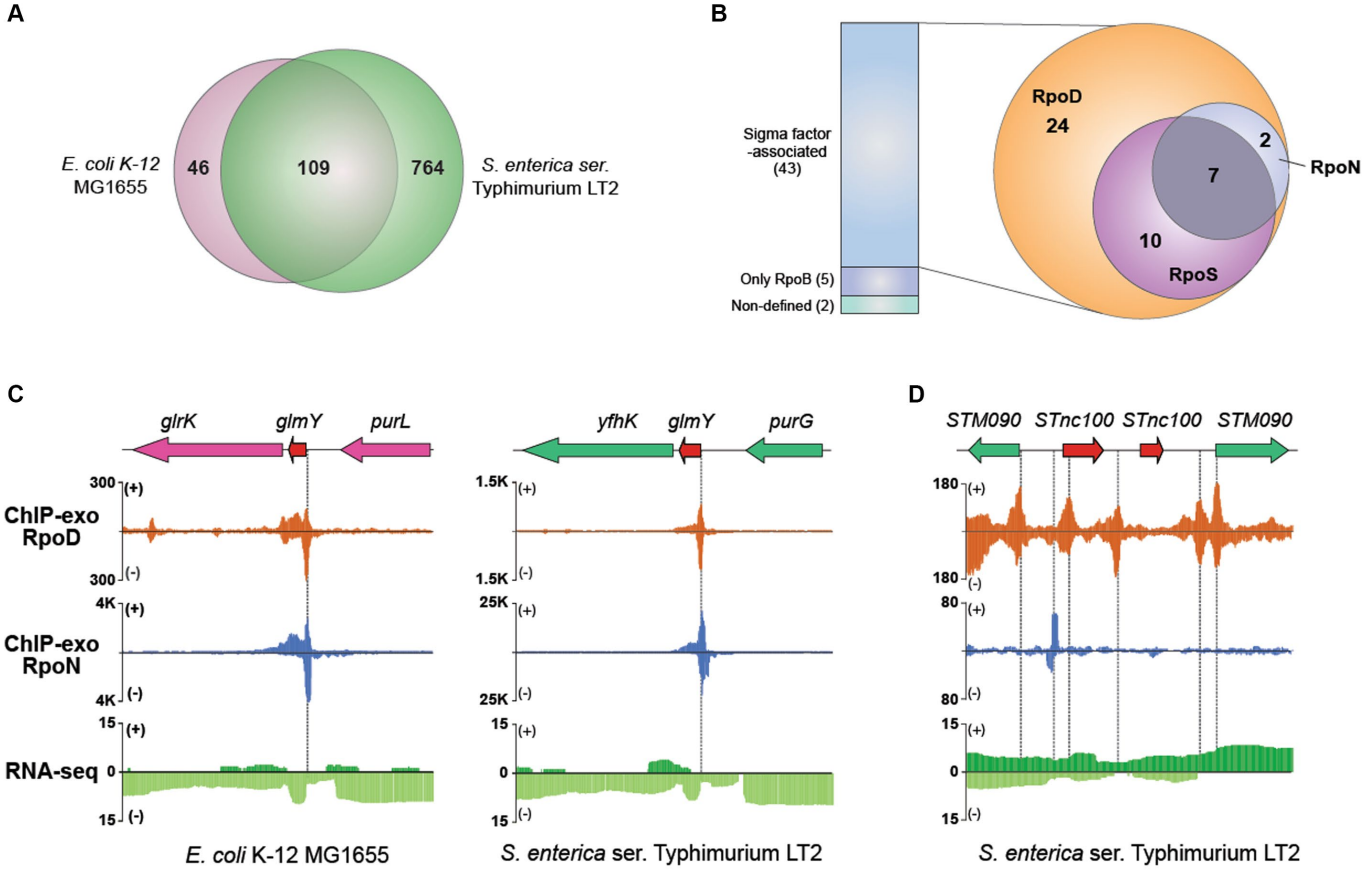
Comparative sigmulon analysis for sRNA between MG1655 and LT2. **(A)** Venn diagram for the number of orthologous sRNAs between MG1655 and LT2. **(B)** The binding events of sigma factors (RpoD, RpoN, and RpoS) on expressed sRNA. **(C)** Zoomed-in example of an orthologous sRNA (*glmY*) showing the overlapped RpoD and RpoN binding sites both in MG1655 and LT2. **(D)** A representative example of sRNA (*STnc100*) with multiple copies in the LT2 genome and its binding pattern of RpoD and RpoN.

Using this approach, 873 sRNAs (chromosome: 868; plasmid: 5) have been compiled into the final dataset for further analysis (Supplementary Table S5). Among them, 109 orthologous sRNAs between MG1655 and LT2 were predicted, leaving 764 sRNAs being unique for LT2 (Figure 6A). For example, *RyeF (micL)* and *micA* (*sraD*) have been identified as typical non-coding RNAs in both *E. coli* and *Salmonella* that is important for enduring stress from cell wall damage (Peschek et al., 2019; Ponath et al., 2022). The percentages of sRNAs distribution on forward (805/873) and reverse strands (68/873) were 92.2% and 7.79%, respectively.

Subsequently, to characterize the detailed features of each sRNA, we first verified their expression through RNA-seq data and further investigated binding events of RpoB and sigma factors using ChIP-exo for those whose expressions were confirmed (Figure 6B). Out of 50 expressed sRNAs, 42 were identified to have their bound sigma factors. Interestingly, all sRNAs were found to be bound by RpoD. Among these, 17 sRNAs were bound by RpoS as well, while nine sRNAs were co-bound by RpoN. Additionally, seven sRNAs (*ffs*, *STnc700*, *glmY*, *csrB*, *STnc3150*, *sraF*, *Spot42_spf*) were found to be bound by all three sigma factors. Figure 6C presents a representative example of the sRNA (*glmY*), which is bound by both RpoD and RpoN. The promoter region of *glmY* is highly recognized by the various sigma factors, not only in LT2 but also in MG1655. The binding sites of RpoD and RpoN are overlapping, indicating that two sigma factors would compete with each other to occupy this promoter. Figure 6D illustrates representative promoter regions of STnc100, which have multiple copies in the LT2 genome.

## Discussion

Promoters, the fundamental DNA motifs where general transcription factors and the polymerase bind, play as an “on-switch” initiating transcription. Therefore, its identification is an important event in unveiling the genomic and transcriptomic properties of living organisms. For instance, in pathogen bacteria, characterization of active promoters under infection-relevant conditions is required for understanding its pathogenicity. Herein, we shed light on the uncharted territory of the genome-wide promoter map of LT2 by employing ChIP-exo for RNAP (RpoB) and the four sigma factors (RpoD, RpoN, RpoS, and RpoE), a higher-resolution chromatin immunoprecipitation technique compared to conventional ChIP-chip or ChIP-seq, and integrating them with RNA-seq.

The ChIP-exo experiments for RNAP (RpoB) and the four major sigma factors (RpoD, RpoN, RpoS, and RpoE) uncovered massive binding sites for each factor. Compared to previous related studies, it can be inferred that ChIP-exo may update the sigmulons of LT2. In the case of RpoD, the ChIP-chip method identified 832 RpoD binding regions associated with RpoB in *S.* Typhimurium SL1344 (Kröger et al., 2012). In this study, we identified 980 RpoD binding sites associated with RpoB. Although the application of ChIP-exo to pathogenic strains needs validation, considering that LT2 has 260 fewer genes than *S.* Typhimurium SL1344, it can be suggested that introducing ChIP-exo to *Salmonella* sigmulons research may offer advantages due to its higher resolution. Over one-third (1,752/4,714, 37.2%) of genes or transcription units were identified to RpoD sigmulon. The result is in line with the fact that RpoD has been reported as a primary sigma factor which is crucial for the expression of most housekeeping genes during exponential growth (Gruber and Gross, 2003). The number of RpoN sigmulon could be also updated in the same way. While previous studies identified 186 RpoN binding sites in *S.* Typhimurium 14,028 s (Bono et al., 2017) or 250 RpoN binding sites in closely related species *E. coli* K-12 MG1655 (Bonocora et al., 2015), it was shown that 418 genes were directly regulated by RpoN. In the case of LT2, a previous study utilizing ChIP-chip identified 70 binding sites, and in this study, we confirmed the presence of 63 of these sites (Samuels et al., 2013). Additionally, in the remaining binding sites investigated in our study, we observed the continued presence of the RpoN motif.

Apart from RpoB which is a subunit of RNAP core enzyme, sigma factors compete with each other to recruit the apo-enzyme complex to initiate the transcription process. In addition, several studies suggested that alternative sigma factors might have their own set of promoters to bind (Grigorova et al., 2006; Li et al., 2015; Bono et al., 2017). While a total of 156 overlapping regions bound by RpoD and RpoN were identified, 162 overlapping sites between RpoD and RpoS were observed as well. Given the widespread use of sigmulons to identify promoters, the promoter can be strongly validated when overlapping binding region by several sigma factors. Although our functional assay demonstrated that the sigma factors have their own regulon, 15.8% (276/1,752) of RpoD binding regions exhibited sharing to RpoS and RpoN, which suggests these regions are promoters in common growth conditions with high probability. Furthermore, these results imply that certain genes may be regulated by multiple sigma factors simultaneously, raising questions how these different sigma factors contribute to characteristic changes in gene expression profiles across various conditions. For RpoS and RpoE, this study showed fewer sigmulons than previously reported since the experiment was conducted under the exponential phase which is not an optimal condition for them. RpoS was found out with the highest expression in the stationary phase (Lago et al., 2017). It was reported that 31 RpoE binding sites were identified which are involved in heat shock and oxidative stress responses through the ChIP-seq method (Li et al., 2015). Nevertheless, it is meaningful that we can discriminate between active and inactive promoters of LT2 during the exponential phase. Meanwhile, it is known that there are multiple transcription start sites (TSSs) within a promoter region (Mejia-Almonte et al., 2020). It would be worthwhile to investigate the number of TSS present within the 2,020 promoters identified in this study using methods such as dRNA-seq and compare them with the 3,838 known *S.* Typhimurium TSS from the 4/74 strain (Kroger et al., 2013).

It has been widely known that extensive overlap between promoters bound by RpoD and alternative sigma factors is an essential phenomenon in bacterial transcription, enabling a highly elaborated transcriptional network with flexibility in gene expression under multiple conditions (Wade et al., 2006). For instance, it was reported that the presence of RpoS was responsible for transcriptional repression of some genes in MG1655, by competing for the shared promoters with RpoD (Cho et al., 2014). However, it has been elusive if there is an overlapping between two subgroups of sigma factors: RpoD-family and RpoN. According to the results of LT2 in this study, there are 60 overlapping binding regions between RpoD and RpoN, which indicates possible competition between different subgroups of sigma factors. Meanwhile, the previous study reporting the overlapping binding patterns of RpoD and RpoH revealed that the genes nearby the shared binding sites were associated with bacterial adaptation to extreme environmental conditions (Wade et al., 2006). Furthermore, the overlapping binding sites were predominantly located within genes, rather than their anticipated intergenic regions. Similarly, there were considerably more intragenic RpoN binding sites than intergenic ones in this study as well. Despite the presence of conserved motifs when compared to intergenic regions, these sites exhibited less association with RpoB, implying their distinct biological functionality that requires further in-depth investigation.

Meanwhile, while it has been believed that promoters were located mostly upstream of the target genes in the past (Fitzgerald et al., 2018), however, ChIP techniques started to reveal the intragenic binding site of sigma factors and transcription factors such as RutR in regulating pyrimidine catabolism (Shimada et al., 2008) or FliA (σ28; Fitzgerald et al., 2018) which have *bona fide* intragenic promoters with each being evolutionary strongly selected in MG1655. These findings have proposed intragenic promoter to be functional as being shown non-random behavior of location within a gene. The orientation of RpoN binding sites in bacteria such as MG1655 (63.0%, 85/136) has been described that most of them are intragenic regions (Bonocora et al., 2015). Therefore, in this study, we also focus on intragenic binding sites. Surprisingly, our results have revealed an extensive number of RpoN binding sites, with 72% of them located within intragenic regions, which is congruent with previous studies of closely related strain *S.* Typhimurium 14,028 s (77.4%, 144/186; Bono et al., 2017). In addition, it has been reported that several intragenic RpoN binding sites in *S. enterica* and *E. coli* are conserved which further supports that these intragenic sites might play important biological roles (Wade et al., 2006). While studies have proposed that RpoN intragenic sites could act as roadblocks for transcriptional interference (Bono et al., 2017), additional analysis is needed as of now.

To understand the complex interactions which turn on the transcription process, a thorough investigation of regulatory components in bacterial transcription is required. Since RpoD and RpoN constitute different sigma factor families, the difference lies in the structure which affects the recognition of sequence motifs on the genomic DNA. Since both sigma factors RpoD and RpoN bind to the same site on RpoB, it was presumed that either of the alternative sigma factor bindings would block the other in these sites, indicating direct competition between RpoD and RpoN (Lonetto et al., 1992; Luo and Morrison, 2003). Contrary to the RpoD having the highest affinity to RNAP under the growth condition (Paget and Helmann, 2003) so that holoenzyme with RpoD forming open complex for transcription initiation (Bono et al., 2017), the RpoN-containing holoenzyme binds to DNA in an inactive form and bacterial enhancer-binding proteins are required to activate the complex to begin transcription (Bonocora et al., 2015). Under nitrogen-limiting conditions, RpoN forms a part of the holoenzyme that binds to the promoter region of genes responsible for overcoming this stress. Our analysis demonstrated that RpoN can regulate itself during the exponential phase.

Upon conducting a comparative analysis with MG1655, interspecies conserved binding sites of RpoD and RpoN were discovered. Out of the 3,158 orthologous genes, 71.3% showed the same binding pattern (31 had both sigma factors bound, 991 had RpoD bound, 50 had RpoN bound, and 1,181 had neither factor bound), while the remaining 905 genes (28.7%) were subject to differential transcriptional regulation. The different binding pattern of RpoD and RpoN in the conserved genes reflects the possible diverse transcriptional regulation even in closely related enterobacteria species. As genetic differences have been found on pathogenicity islands between *E. coli* and its closely related strains (Blattner et al., 1997; McClelland et al., 2000), further investigation into the unique genes may provide valuable insights into the genomic contents for pathogenesis. Furthermore, LT2 showed no preference in binding positions of intragenic binding sites. Specifically, 14.6% (67/460) of intragenic RpoD and 11.6% (35/303) of intragenic RpoN were found to contain “ATG” (gene start codon). While RpoN intragenic binding sites were detected at approximately 360 to 760 bases from the gene start in MG1655 (Fitzgerald et al., 2018), the difference between the positions in LT2 was not statistically significant despite the higher number of binding sites for both sigma factors observed toward the gene start.

In case of sRNAs, 871 *S.* Typhimurium sRNAs have been predicted up to date (Kroger et al., 2013; Barnhill et al., 2019; Houserova et al., 2021). Similarly, 873 sRNAs were identified in this study. Then, the integrative investigation of *in silico* computational analysis and *in vivo* experimental genome-wide measurements has explored the landscape of annotated sRNAs in LT2. By examining the expression of sRNAs and their sigma factor-binding patterns, it became possible to update the characteristics of LT2 sRNAs. Among the 873 sRNA candidates in LT2, only 50 sRNAs were found to be expressed, possibly due to the transcriptome analysis being performed only under one growth condition, such that those other sRNA genes were not measured under their activating conditions. Especially, it is well-known that sRNAs are often highly expressed during the stationary phase compared to the exponential phase (Frohlich et al., 2012), and a considerable number of sRNAs are reported to be regulated by RpoS (Metaane et al., 2022). Therefore, it is likely that the expression of sRNAs could have been limited under the mid-exponential phase condition of LT2 used in this study. Additionally, the inclusion of putative sRNA genes without experimental verification could have resulted in false positives. Follow-up studies overexpressing each sigma factor may give direct evidence for their impact on sRNA expression. Despite this, we were able to determine the sigma factors involved in the transcription process for each of the sRNAs that were confirmed to be expressed.

We acknowledge several limitations in this study. First, the LT2 strain used in this study is an attenuated strain which has an avirulent nature by a rare start codon (UUG) for the *rpoS* gene; therefore, it would not be appropriate for studying the pathogenic properties of *S.* Typhimurium. However, the laboratory strain has served as an anchor strain for numerous profound discoveries in the fields of gene regulation and biochemistry of *Salmonella* and other enterobacteria (Samuels et al., 2013; Patel et al., 2020). Second, the current sampling condition may not be optimal for the expression of pathogenicity. Further studies under various infection-relevant conditions, such as inducing environmental shock (Kroger et al., 2013), will provide an opportunity to deepen our understanding of the pivotal roles of sigma factors in *Salmonella* pathology. Similarly, sRNAs identified in this study are needed to be validated by northern blot or Hfq coimmunoprecipitation coupled with RNA-seq (Hfq-coIP-seq) under various conditions. Nevertheless, this study demonstrated the feasibility of a methodology that integrates ChIP-exo and RNA-seq to explore the genome-wide mapping of LT2 sigmulons. This approach allowed us to obtain high-resolution information on the binding locations of sigma factors at the genome-wide level through ChIP-exo, as well as confirm gene expression profiles using RNA-seq. One notable advantage is that it enables us to uncover the physical characteristics of promoters, including precise binding locations and lengths, which were not achievable with traditional methods such as dRNA-seq (Kroger et al., 2013) or TSS-seq (Vera et al., 2020), commonly used to identify the presence of promoter regions. When combined with knock-out or overexpression techniques, benchmarking some sigma factor studies in *Corynebacterium* (Dostalova et al., 2017; Toyoda and Inui, 2018) can provide more detailed information about the LT2 sigmulon and sigma factor-associated sRNA expression patterns. Moreover, this approach can be extended to other pathogenic *Salmonella* strains such as 14,028 s, SL1344, SL1344, ST19 strain 4/74, and ST313 strain D23580, which investigated their genomic characteristics such as promoter regions and sRNA encoding regions by TSS-seq, differential RNA-seq, or Hfq-coIP-seq (Kröger et al., 2012; Kroger et al., 2013; Srikumar et al., 2015; Hammarlof et al., 2018). Particularly, by examining distinctive patterns of the sigma factor network under various conditions, such as a time-course study to investigate growth phase-specific infectivity (Lee and Falkow, 1990; Russell et al., 2004; Fan et al., 2019), responses to various infection-relevant environmental shocks (Kroger et al., 2013), or dynamics observed during actual infection scenarios (Srikumar et al., 2015), an unprecedented understanding of the complex regulatory mechanism of the network could be achieved. Other transcription factors, such as OmpR whose binding sites and regulons in *S.* Typhimurium have been previously investigated using ChIP-chip (Quinn et al., 2014) and ChIP-seq (Perkins et al., 2013), can have their regulons updated with higher resolution and conducted comparative analysis with other species (e.g., *E. coli*; Chakraborty and Kenney, 2018) or strains to explore strain-specific characteristics.

Collectively, using experimental results of ChIP-exo with near single base-pair resolution, the detailed genome-wide promoter map of LT2 became available to facilitate further in-depth experiments and genomic analysis. Especially, integration with the transcriptome profiling provided unprecedented information about the genomic and transcriptomic features of the sigma factor network in *S.* Typhimurium. These findings suggest that the combinational analysis using ChIP-exo and RNA-Seq can offer a better understanding of promoter regions and their functionality, on the genome-scale level, and hence would provide insights into how bacteria modulate transcriptional regulation in response to environmental changes.

## Materials and methods

### Bacterial cell culture

The studied bacterium LT2 stored at −80°C in the form of the 50% (v/v) glycerol stock was inoculated into M9 minimal medium (47.8 mM Na_2_HPO_4_, 22 mM KH_2_PO_4_, 8.6 mM NaCl, 18.7 mM NH_4_Cl, 2 mM MgSO_4_, and 0.1 mM CaCl_2_) supplemented with 0.2% (w/v) glucose for seed culture. After incubating overnight at 37°C in a shaking incubator (200 rpm), the resulting culture was used to inoculate the fresh media for growth curve measurement with three biological replicates. For ChIP-exo and RNA-seq, the fresh culture was incubated to the mid-log phase (OD_600_ ≈ 0.5), and samples were prepared according to each protocol from the same flask. These samples can be stored at −80°C at the step mentioned in each protocol.

### ChIP-exo experiment

The binding maps of RNA polymerase RpoB subunit and RpoD, RpoN, RpoS, and RpoE sigma factor candidates *in vivo* were recognized by using ChIP-exo method previously described (Seo et al., 2014) with modification. In brief, formaldehyde crosslinking is employed to LT2 cells, and the DNA segments with bound protein candidates by chromatin immunoprecipitation (ChIP) were isolated to prepare the samples. The crosslinking cells can be stored at −80°C until further use. Then, several specific antibodies (1,5,000 dilution) identifying RpoB subunit (WP002; Neoclone), RpoD (WP004; Neoclone), RpoN (W0005; Neoclone), RpoS (WP009, Neoclone), and RpoE (WP007, Neoclone) were treated, followed by continuously rigorous washings.

The remaining steps for the ChIP-exo method include the usage of chromatin beads to conduct on-bead enzymatic reactions with modifications (Rhee and Pugh, 2011, 2012). At first, the cut DNA from chromatin–beads complex was repaired by using the NEBNext End-Repair Module (New England Biolabs), which subsequently added a single dA overhang by NEBNext dA-Tailing Module (New England Biolabs). Then, the 5′-phosphorylated first adaptor was ligated by NEBNext Quick Ligation Module (New England Biolabs), and PreCR™ Repair Mix (New England Biolabs) was used to manage nick repair. The chromatin was treated with lambda exonuclease and RecJ_f_ exonuclease to become eluted from the beads, followed by a step of incubation at 65°C to reverse the cross-linked the interested protein–DNA complex. Attached RNAs and proteins were removed, and the DNA samples were prepared for primer extension treated with dA-tailing and ligation of the second adaptor by NEBNext Quick Ligation Module (New England Biolabs). DNA purification step was conducted with GeneRead Size Selection Kit (Qiagen). Then, polymerase chain reaction (PCR) was executed using Phusion High-Fidelity DNA Polymerase (New England Biolabs) to amplify the DNA sample which was purified by the same GeneRead Size Selection Kit (Qiagen) and quantified using Qubit dsDNA HS Assay Kit (Life Technologies). The DNA sample quality was checked using Agilent High Sensitivity DNA Kit using Agilent 2100 Bioanalyzer (Agilent); then, the sample was sequenced by MiSeq (Illumina) according to the instructions. ChIP-exo experiments were conducted in biological duplicate.

### RNA-seq expression profiling

To prepare samples for RNA-seq, bacterial cells were cultured until they reached the exponential phase. Next, a 2-fold volume of RNAprotect™ Bacteria Reagent (Qiagen) was mixed with the 2 mL cells and immediately vortexed for 5 s. After 5 min of incubation at room temperature, the pellet was harvested by centrifugation with 5,000 × g for 10 min and fully removed the supernatant, which can be stored at −80°C until further use. Total RNA, including sRNAs, samples were isolated with RNeasy® Plus Mini kit (Qiagen) and quantified by NanoDrop 1000 spectrophotometer (Thermo Scientific). The quality was checked using RNA 6000 Pico Kit using Agilent 2100 Bioanalyzer (Agilent). Paired-end, strand-specific RNA-seq library was built using the KAPA RNA Hyper Prep kit (Kapa Biosystems) according to instructions. The obtained libraries were analyzed on an Agilent Bioanalyzer DNA 1000 chip (Agilent) and sequenced by MiSeq (Illumina). Two biological replicates were used to prepare samples, sequenced, and used to acquire TPM values for each gene.

### Peak calling for ChIP-exo dataset

The peak calling process was conducted as previously described (Seo et al., 2014). In brief, ChIP-exo sequence reads were mapped onto the reference genome (chromosome: NC_ 003197.2; plasmid: NC_003277.2) using Bowtie and its default options to produce SAM output files. To filter out the false-positive peaks, peaks with signal-to-noise (S/N) ratios less than 1.5 were removed. The noise level was set to the top 5% of signals at genomic positions as this represents the background level plateau. The intensities of the top 5% signals from each ChIP-exo replicate for each condition were correlated well with the total number of reads. The calculation method for the S/N ratio is similar to that for ChIP-chip peak intensity which the immunoprecipitated signal is divided by the Mock signal. Subsequently, each peak was assigned to the nearest gene. The whole peak positions in the genome-scale level were visualized with the Metascope (Bang et al., 2023b), and further curation step to minimize the false peaks was carried out.

### Motif search from ChIP-exo peaks

From the peak calling outputs, the discovery search for sequence motifs of sigma factors was conducted using MEME from the MEME suite (Bailey et al., 2009) with default settings. The binding motifs were calculated from at least 90% of the input sequences. The reference genome (chromosome: NC_003197.2; plasmid: NC_003277.2) was used for the extraction of the sigma factor binding region sequences.

## Data availability statement

The data presented in the study are deposited in the NCBI GEO repository, accession number GSE119967 (https://www.ncbi.nlm.nih.gov/geo/GSE119967).

## Author contributions

S-ML: Conceptualization, Data curation, Formal analysis, Investigation, Methodology, Visualization, Writing – original draft, Writing – review & editing. HL: Data curation, Methodology, Writing – review & editing. AT: Data curation, Methodology, Writing – review & editing. LN: Writing – review & editing. JP: Writing – review & editing. E-JL: Writing – review & editing. BP: Writing – review & editing. DK: Conceptualization, Funding acquisition, Project administration, Writing – review & editing.

## Footnotes

^1^ https://www.ncbi.nlm.nih.gov/nuccore/NC_000913.3

^2^ https://ecocyc.org/

^3^ http://kwanlab.bio.cuhk.edu.hk/BSRD

^4^ http://rfam.xfam.org
