# Supplementary figures and images for: Experimental promoter identification of a foodborne pathogen Salmonella enterica subsp. enterica serovar Typhimurium with near single base-pair resolution
Source: Front Microbiol. 2024 Jan 4;14:1271121. doi: 10.3389/fmicb.2023.1271121 (PMC10794520; doi:10.3389/fmicb.2023.1271121)

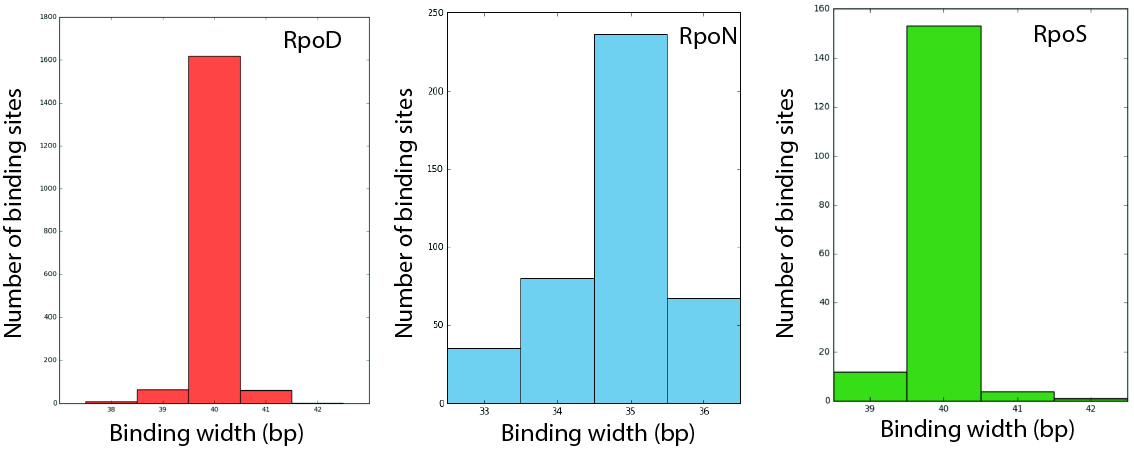

Supplement: Supplementary file 1 [file Image_1.TIF]
